# Investigation of Spatial and Temporal Trends in Water Quality in Daya Bay, South China Sea

**DOI:** 10.3390/ijerph8062352

**Published:** 2011-06-22

**Authors:** Mei-Lin Wu, You-Shao Wang, Jun-De Dong, Cui-Ci Sun, Yu-Tu Wang, Fu-Lin Sun, Hao Cheng

**Affiliations:** 1 State Key Laboratory of Oceanography in the Tropics, South China Sea Institute of Oceanology, Chinese Academy of Sciences, Guangzhou 510301, China; E-Mails: mlwu@scsio.ac.cn (M.-L.W.); dongjunde@vip.163.com (J.-D.D.); suncuici722@tom.com (C.-C.S.); wangyutu@163.com (Y.-T.W.); flsunch@yahoo.cn (F.-L.S.); chenghao@scsio.ac.cn (H.C.); 2 Marine Biology Research Station at Daya Bay, Chinese Academy of Sciences, Shenzhen 518121, China

**Keywords:** Daya Bay, principal component analysis, multidimensional scaling analysis, statistical techniques, water quality

## Abstract

The objective is to identify the spatial and temporal variability of the hydrochemical quality of the water column in a subtropical coastal system, Daya Bay, China. Water samples were collected in four seasons at 12 monitoring sites. The Southeast Asian monsoons, northeasterly from October to the next April and southwesterly from May to September have also an important influence on water quality in Daya Bay. In the spatial pattern, two groups have been identified, with the help of multidimensional scaling analysis and cluster analysis. Cluster I consisted of the sites S3, S8, S10 and S11 in the west and north coastal parts of Daya Bay. Cluster I is mainly related to anthropogenic activities such as fish-farming. Cluster II consisted of the rest of the stations in the center, east and south parts of Daya Bay. Cluster II is mainly related to seawater exchange from South China Sea.

## 1. Introduction

Daya Bay lies on the southern coast of China (from 22°31′12″ to 22°50′00″ N, 114°29′42″ to 114°49′42″ E) like a door shape, with a coastal line of 92 km, and an area of 600 km^2^. The water depth ranges from 6 to 16 m, with an average water depth of 10 m [1]. There are two towns (Dapeng Town and Nanao Town) in the western coast area. The north and east coast area belong to Huizhou, China, there are five towns, namely Xiachong Town, Aotou Town, Renshan Town, Xunliao Town and Tiechong Town. In recent years, the rapid economic development and anthropogenic activities from Shenzhen and Huizhou have had a great influence on the environment of this bay. It has been one of the main aquaculture areas in Guangdong Province, due to its excellent water quality. Daya Bay Nuclear Power Station (DNPS) and Lingao Nuclear Power Station (LNPS) have also been operated there. The stronger northeast monsoon prevails in the area from October to April; and the Southeast Asian monsoons predominate southwesterly from May to September.

Some studies have been conducted on the environmental effects caused by anthropogenic activities in the bay. Some authors have described phytoplankton biomass and primary production [2], high temperature effluent from DNPS [3], nutrients in aquaculture area [4] and nutrients [5–8]. Other studies have been focused on water quality [9–11], organic pollutants in water or surface sediments [12], and heavy metals [13,14].

In the present work, we have done a relatively comprehensive study of the water quality of Daya Bay. Besides, multivariate statistical techniques were used to establish the effects caused by the different human activities and seasonal variations found in the bay.

## 2. Materials and Methods

### 2.1. Sampling and Analytical Methods

**Figure 1.**
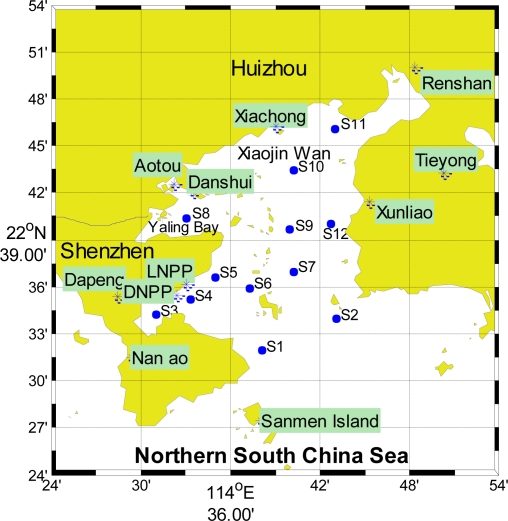
Monitoring stations in Daya Bay [10].

Seawater samples for analysis of nutrients, chlorophyll *a* were taken using 5-L GO FLO bottles at surface and bottom layers of all stations (Figure 1) in January (Winter), April (Spring), August (Summer) and November (Autumn) in 2003 (GB12763-91, China). The determinations of water temperature, and salinity were performed *in situ* by the water quality Monitoring System. Water samples from various depths were analyzed for nitrate and nitrite with a SKALAR auto-analyzer (Skalar Analytical B.V. SanPlus, The Netherlands). Ammonium and total phosphorus (TP) were analyzed by the methods of oxidation with hypobromite and molybdophosphoric blue using an UV1601 spectrophotometer (Shimadzu Corporation, Japan), respectively. Chemical oxygen demand (COD), 5-day bio-chemical oxygen demand (BOD_5_) and transparency (Secchi) were tested according to “The specialties for marine monitoring” (GB17378.4-1998, China) and Wang *et al.* [8]. Dissolved oxygen (DO) was determined with the Winkler titration method. DIN is the bsum of nitrate and nitrite and ammonium.

Two replicates of 1.5 L samples from the depths mentioned above were filtered through 47 mm GF/F filters and were deep frozen immediately at −20 °C. At the end of the cruise, all filters were transported to the shore laboratory in liquid nitrogen. Within a week, the chlorophyll *a* (Chl *a*) was extracted in10 mL 90% acetone in the dark for 24 h in a refrigerator and its concentration was determined with 10-AU Fluorometry (Turner Designs, USA).

### 2.2. Data Treatment

The data for this study are the mean data obtained from surface and bottom waters. In this study, well known multivariate statistical techniques such as principal component analysis (PCA) and multidimensional scaling (MDS) were used to identify the anthropogenic and natural effects on the water environment status. PCA was used to understand the correlation structures between variables and to combine them into groups to reduce the dimensions of the variables for making decisions and interpretations easier. MDS analysis was used to explore the similarities or dissimilarities (distances) in water quality between the surface water quality monitoring stations in the bay.

Data was auto-scaled in order to avoid misclassification due to wide differences in data dimensionality. The data were normalized with mean and variance of zero and one, respectively. All the procedures were performed using MATLAB R 2008b (Mathworks Inc., USA).

## 3. Results

Two-way analysis of variance (ANOVA) was performed in order to show the existence of temporal and spatial differences between water samples from Daya Bay. Results from ANOVA did not show any temporal variations of COD obtained from different sampling campaigns but showed significant temporal change of other variables, such as temperature, salinity, Secchi, DO, BOD_5_, Chl *a*, DIN and TP. Significant spatial differences were found for all the variables including temperature, salinity, Secchi, DO, BOD_5_, COD, Chl *a*, DIN and TP. This result may be explained by human activities and natural influences.

**Figure 2.**
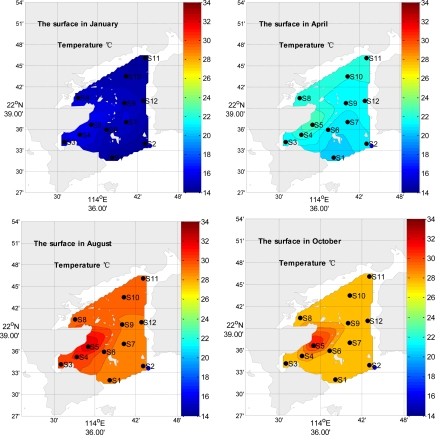
The spatial distribution of surface temperature in four seasons, respectively.

**Figure 3.**
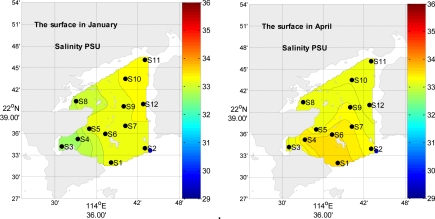

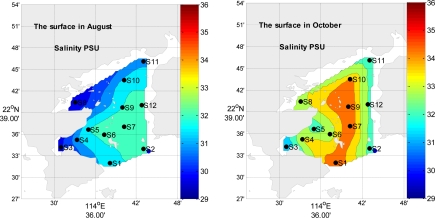
The spatial distribution of surface salinity in four seasons, respectively.

The horizontal distributions of the surface temperature are similar in all four seasons, with higher values in the western and north parts of the bay than in other parts of the bay (Figure 2). The horizontal distributions of the surface salinity are similar in four seasons, the surface salinity decrease from the mouth to the top in the bay in summer; it is higher in the eastern and northern part than that in western part in winter (Figure 3). In summer, the salinity is lower than that in other seasons; the rain is an important factor, which diminishes the surface salinity.

### 3.1. Principal Component Analysis

The particular challenge in the case of water quality monitoring is the complexity associated with analyzing the large number of measured variables. Therefore, in this study, principal component analysis, which is a well known data reduction technique, was utilized to extract the main components of the water quality data.

The loadings of the three retained principal components (PCs) with classical PCA are shown in Table 1. PC1 (35.1039% of the variance) is mainly related to Secchi, BOD_5_, COD and DIN. PC2 (15.3675% of the variance) is characterized by Chl *a* and TP. PC3 explains 15.3675% of the variance, related mainly to DO.

**Figure 4.**
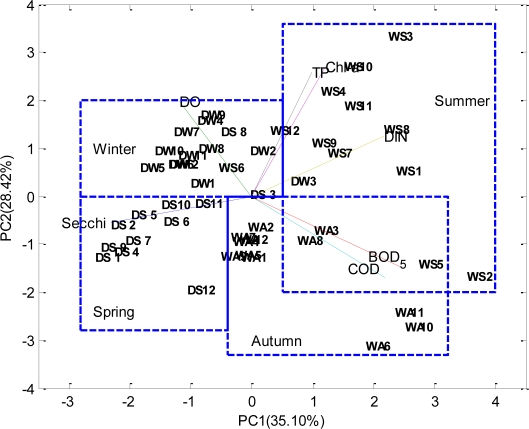
The loadings of variables and scores of the 12 stations for the first two PCs (DW-Winter, DS-Spring, WS-Summer and WA-Autumn), respectively. The number denotes the station number; the letter denotes the variable.

The matrix of scores provides information about the distribution of patterns or sources of contamination among samples. The matrix of loadings defines the contribution of the original variables to each one of these contamination patterns or sources. The data are distributed in a limited region of space spanned by the two well-defined PC axes. Seasonal pattern has been demonstrated by the scores of the twelve stations in four seasons. The seasonal stations are separated in the plane; the seasonal character has distinct. The border of the four groups is clearly marked in Figure 4. The principal components score plot can not only interpret the spatial distribution by clustering the samples, but can also describe their different characteristics and help to find out the relationship between different parameters (variables) by the parameter lines.

The parameter lines were obtained from the factor loadings of the original variables. They stand for the contribution (significance) of the parameters to the samples. The closer two-parameter lines lie together, the stronger the mutual correlation is. For example, TP and Chl *a* have the highest positive correlation coefficient (0.95), so the two lines are very close. A very weak or no correlation between the two parameters was found, angle between two lines was about 90°. Similarly, an obtuse angle between two lines represents a negative correlation, *etc.*, DO and Chl *a*.

From the principal components score plot, the temporal variations of the samples in the bay can be clearly observed. In the first quadrant, a cluster of the samples in winter is characterized by high TP, DIN and Chl *a*, as these parameter lines are located in this quadrant. As mentioned before, external pollution sources from land-resources could be introduced into the bay by river, runoff and rain, *etc.*

### 3.2. Multidimensional Scaling Analysis

In this section, the water quality characteristics data were analyzed by using two-dimensional and three-dimensional MDS analysis to identify similarities and differences between surface water quality monitoring stations. STRESS (Standardized Residual Sum of Squares), which is used to evaluate how well a particular configuration reproduces the observed distance matrix, was found to be 0.00008 even for two-dimensional MDS. Because, STRESS values close to zero shows that the “fit” is almost perfect and the results of the MDS analysis is reasonable and reliable.

**Figure 5.**
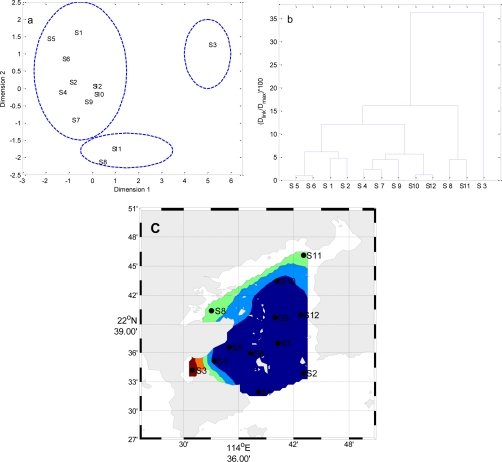
The results of multidimensional scaling analysis and cluster analysis: (**a**) Multidimensional scaling analysis plot for the monitoring stations in Winter; (**b**) Dendrogram based on Ward’s method for monitoring stations in Winter; (**c**) Map of the resulting zones of Daya Bay in Winter.

**Figure 6.**
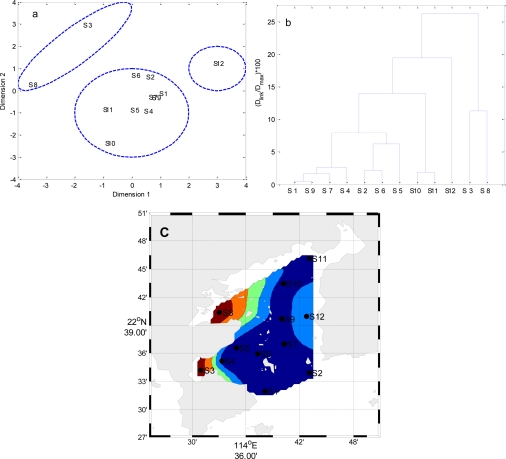
The results of multidimensional scaling analysis and cluster analysis: (**a**) Multidimensional scaling analysis plot for the monitoring stations in Spring; (**b**) Dendrogram based on Ward’s method for monitoring stations in Spring; (**c**) Map of the resulting zones of Daya Bay in Spring.

**Figure 7.**
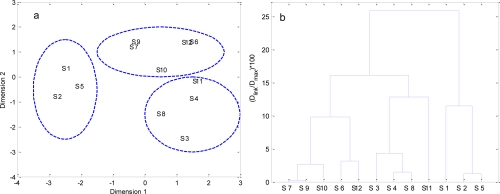

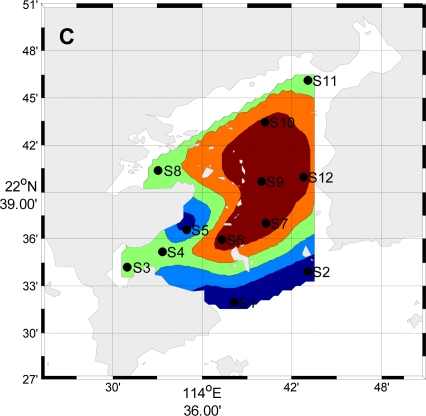
The results of multidimensional scaling analysis and cluster analysis: (**a**) Multidimensional scaling analysis plot for the monitoring stations in Summer; (**b**) Dendrogram based on Ward’s method for monitoring stations in Summer; (**c**) Map of the resulting zones of Daya Bay in Summer.

**Figure 8.**
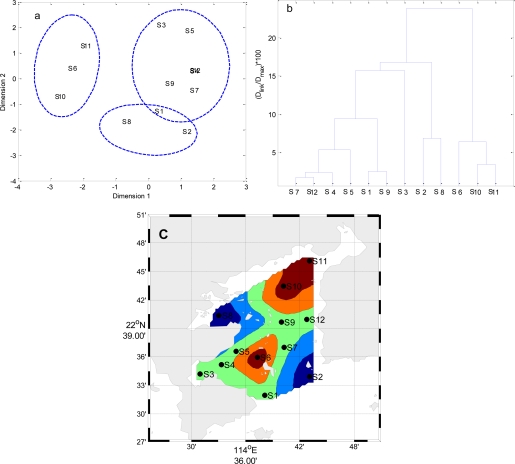
The results of multidimensional scaling analysis and cluster analysis: (**a**) Multidimensional scaling analysis plot for the monitoring stations in Autumn; (**b**) Dendrogram based on Ward’s method for monitoring stations in Autumn; (**c**) Map of the resulting zones of Daya Bay in Autumn.

**Figure 9.**
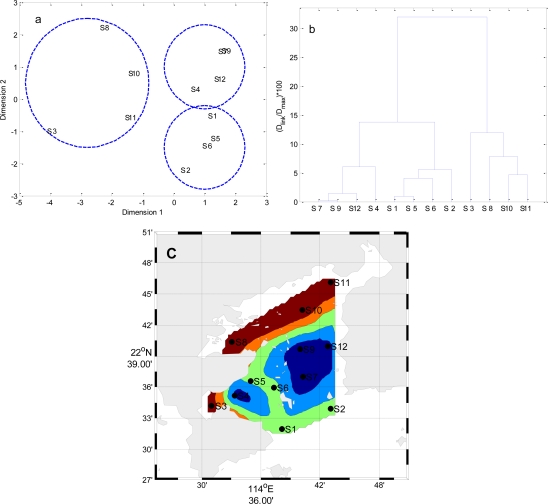
The results of multidimensional scaling analysis and cluster analysis: (**a**) Multidimensional scaling analysis plot for monitoring stations in overall spatial pattern; (**b**) Dendrogram based on Ward’s method for monitoring stations in overall spatial pattern; (**c**) Map of the resulting zones of Daya Bay in overall spatial pattern.

Temporal MDS was performed on raw data after dividing the whole data set into four (winter, spring, summer and autumn) seasonal groups (Figures 5 to 8). The 12 surface water quality monitoring stations according to Dimension-1 and Dimension-2 via MDS analysis had different spatial patterns in four seasons, respectively (Figures 5 to 8). In Winter, where all the 12 sampling sites on the bay were grouped in to three clusters with cluster 1 with grouping four sites (S1, S2, S4–S7, S9, S10 and S12), cluster 2 with another two sites (S8 and S11), and cluster 3 comprising site (S3). The circulation has different characteristics in different seasons, and land-resources inputs have been different. Thus, MDS was performed on the mean values of variables. The 12 surface water quality monitoring stations were grouped into two clusters (Figure 9). Cluster I consisted of S3, S8, S10 and S11 in the west and north coastal parts of Daya Bay, and cluster II consisted of the rest stations in the center, east and south parts of Daya Bay.

## 4. Discussion

Daya Bay is a semi-closed bay and has several seasonal creeks. In the south, Daya Bay exchanges its water with the South China Sea. Therefore, the water in South China Sea plays an important role on that in Daya Bay. Furthermore, Daya Bay is located in a subtropical zone, where the stronger northeast monsoon prevails from October to April; and the southwesterly Southeast Asian monsoons predominate from May to September. The seasonal change character has been proved the dry and wet seasonal characters in Daya Bay [7]. The average precipitation is 1,827 mm in Daya Bay, the maximum rainfall is 370 mm in June, and the minimum rainfall is 30 mm in December [15]. There is plenty of rain from May to October (about 80% precipitation of the whole year), and there is less from November to the next April (about 20% precipitation of the whole year). The salinity presents the most significant temporal differences (*p* = 0.00). In Summer, fresh water input from several seasonal rivers diminishes the surface salinity, with lower salinity in west north part of the bay. The salinity may be strongly related to the runoff and precipitation.

Temperature has a distinct seasonal change (Figure 2).Temperature is an important climatic factor, it can be an important indicator of natural processes in the subtropical area [7]. The result of analysis of variance analysis also shows that the temperature presents the most significant temporal difference (*p* = 0.00). The spatial distribution of the water temperature showed high values in the western part of Daya Bay near the nuclear power stations (near S5) and low values in the eastern and southern parts of the Bay. Warm discharge from DNPS could lead to the disadvantaged growth of some marine species relative to others [16]. With a 1∼3 °C change of temperature, the discharge plume from DNPS may also result in some ecological changes in the lower portion of Daya Bay, particularly in Dapeng Cove [3].

The stratification due to temperature and salinity differences between surface and bottom waters within the Bay started to develop in June, was strongest from July to September, and disappeared in October. The water of lower temperature and high salinity intrudes into the bay along the bottom from the South China Sea under the influence of the weak monsoons southwesterly from May to September [15,17]. The stratified waters were distinct in Summer. Temperature and salinity in the Bay were uniform with depth from November until April of the following year [18]. The waters are vertically mixed in Daya Bay under the influence of the northeast monsoon [19,20]. In addition, the circulation have different pattern all through year. The clockwise Euler Residual Current in Spring, Summer and Autumn in Daya Bay [1] carried the nutrients from land-based water from the west and north in the bay through this area, meanwhile, tidal current carried the nutrients from the South China Sea through this area as well, which may cause the higher concentration of Chl *a* in this area [5]. The highest phytoplankton density was at station S8 near Aotou harbor and the cage culture areas in the north-western part of Daya Bay. This result is consistent with the higher nutrient levels near station S3 and S8. Nutrients could play an important role in determining the density of phytoplankton in Daya Bay [18]. The mean concentrations of Chl *a* in monitoring stations (S3, S8, S10 and S11) and the rest stations (S1, S2, S4–S7, S9 and S12) are 4.70 mg·L^−1^ and 2.29 mg·L^−1^, respectively. The west and north part of the bay (Dapeng Cove and Aotou) may have higher nutrient concentration than elsewhere [21]. The nutrients play an important role on the water quality in the marine aquaculture area and anthropogenic sources of pollution (S3, S8, S10 and S11).

From the results of the analysis of water balances during both two monsoons periods by box models and mass conservation arguments, the water residence time is shorter during the southwest monsoon period than during the northeast monsoon period [1]. Because the turnover time of seawater within the system favors the operation of different internal processes (e.g., biogeochemical, nutrient assimilation, sediment resuspension) in the water column and changes its own characteristics [22].

Annually, the two clusters have been identified by MDS and CA (Figure 9). The monitoring stations (S3, S8, S10 and S11) in the west and north coastal parts of Daya Bay are together clustered. The rest stations (S1, S2, S4–S7, S9 and S12) in the center, east and south parts of Daya Bay are grouped. The S3 and S8 lie in Dapeng Cove and the northwest part near Aotou harbor, respectively. These two bays are used for fish-farming. The human being activities have a strong influence on aquatic environment in the west and north coastal parts of Daya Bay [23]. The mean concentrations of BOD_5_ in monitoring stations (S3, S8, S10 and S11) and the rest stations (S1, S2, S4–S7, S9 and S12) are 1.73 mg·L^−1^ and 1.28 mg·L^−1^, respectively. The mean concentrations of COD in monitoring stations (S3, S8, S10 and S11) and the rest stations (S1, S2, S4–S7, S9 and S12) are 1.04 mg·L^−1^ and 0.80 mg·L^−1^, respectively. The concentrations of the parameters related to anthropogenic pollution like BOD_5_ and COD are higher in monitoring stations (S3, S8, S10 and S11) than that in the rest stations. This result indicates that contamination is mainly from coast human activities and marine aquiculture. The water exchange is also crucial for the balance between continental inputs delivered to the coastal region and coastal water inflow. The Southeast Asian monsoons, northeasterly from October to April and southwesterly from May to September, have important effects on water quality in Daya Bay.

## 5. Conclusions

In this study, the surface water quality in the Daya Bay was evaluated using the well known multivariate statistical methods PCA and MDS. Two factors explaining the 63.5257% of the total variance in the water quality data were identified. They were termed as nutrient factor and the biological factor. The nutrient factor explained 35.1039% and the agricultural use factor explained 28.4218% of the observed variance in the water quality data. The results of the PCA showed that urban wastewater and agricultural drainage waters were the main sources of the contamination in the bay. According to the Dimension 1, the monitoring stations (S3, S8, S10 and S11) were found to be the most dissimilar stations. These results may provide a basis for taking preventive action to reduce the pollution sources caused from aquiculture and human activities in the bay.

## Figures and Tables

**Table 1.** Loadings of 7 physicalechemical parameters on the seven PCs.

	**PC1**	**PC2**	**PC3**	**PC4**	**PC5**	**PC6**	**PC7**
**Secchi**	−0.4636	−0.1119	−0.2607	0.2654	−0.7799	0.1246	0.1021
**DO**	−0.2384	0.3932	−0.6293	0.1145	0.2366	−0.5657	−0.0585
**BOD_5_**	0.5016	−0.3028	−0.3602	0.2290	0.0282	−0.0341	0.6874
**COD**	0.4370	−0.3401	−0.4570	−0.2205	−0.2120	0.0193	−0.6250
**Chl*a***	0.2433	0.5423	−0.1815	0.4548	0.0705	0.6141	−0.1577
**DIN**	0.4332	0.2497	0.3955	0.3629	−0.4038	−0.5310	−0.1295
**TP**	0.1971	0.5187	−0.0898	−0.6905	−0.3493	0.0620	0.2851
**Eigenvalue**	2.4573	1.9895	1.0757	0.6068	0.5364	0.2769	0.0572
**Variance (%)**	35.1039	28.4218	15.3675	8.6692	7.6634	3.9563	0.8179
**Cumulative (%)**	35.1039	63.5257	78.8932	87.5624	95.2258	99.1821	100.0000
